# Clinical Genetics of Alzheimer's Disease

**DOI:** 10.1155/2014/291862

**Published:** 2014-05-13

**Authors:** Zhangyu Zou, Changyun Liu, Chunhui Che, Huapin Huang

**Affiliations:** Department of Neurology, Fujian Medical University Union Hospital, Fuzhou 350001, China

## Abstract

Alzheimer's disease (AD) is the most common progressive neurodegenerative disease and the most common form of dementia in the elderly. It is a complex disorder with environmental and genetic components. There are two major types of AD, early onset and the more common late onset. The genetics of early-onset AD are largely understood with mutations in three different genes leading to the disease. In contrast, while susceptibility loci and alleles associated with late-onset AD have been identified using genetic association studies, the genetics of late-onset Alzheimer's disease are not fully understood. Here we review the known genetics of early- and late-onset AD, the clinical features of EOAD according to genotypes, and the clinical implications of the genetics of AD.

## 1. Introduction

Alzheimer's disease (AD), the most common progressive neurodegenerative disease and the most common form of dementia in the elderly, results from irreversible loss of neurons, particularly in the cortex and hippocampus. Clinically, AD is characterized by progressive impairments of memory, judgment, decision making, orientation to physical surroundings, and language. Pathologically, AD is characterized by the presence of extracellular neuritic plaques (NPs) containing the *β*-amyloid peptide (A*β*) and neurofibrillary tangles (NFTs) composed of hyperphosphorylated tau protein in the brain.

In 1906, Alois Alzheimer reported the case of a woman who presented with a “peculiar” dementia at the age of 51 years. Alzheimer correlated the woman's cognitive and behavioral features with histopathological findings of extracellular “miliary foci” (senile plaques) and fibrils inside the neurons (neurofibrillary tangles) in her cerebral cortex. Emil Kraeplin subsequently named this presenile (onset <65 years) dementia “Alzheimer's disease” (AD) [1]. Familial clustering of AD was first reported by Sjogren et al. in 1952 [2]. Since the 1980s, various families with AD exhibiting an autosomal dominant pattern of inheritance have been reported [3, 4].

With the advance of molecular genetics, in particular genetic linkage studies, positional cloning, and genome-wide association studies (GWAS), searches for the genes responsible for both autosomal dominant forms of AD and sporadic AD have been widely performed. In this review, we outline the genetic landscape for the disease. We describe what is known about the genetics of AD, the clinical features of early-onset AD (EOAD) according to genotypes, and the clinical implications of the genetics of AD.

## 2. Genetic Epidemiology of AD

AD is classified into two subtypes according to the age of onset. About 1–5% of AD cases present with an early-onset (before the age of 65 years, typically in the late 40s or early 50s) and are classified as having EOAD, whereas >95% of patients develop the disease after the age of 65 years and are classified as having late-onset AD (LOAD) [5]. Family aggregation in AD is more obvious among EOAD patients, who usually show a Mendelian autosomal dominant pattern of inheritance (accounting for <1% of AD cases). However, heritability of AD was estimated to be up to 79% based on twin and family studies [6]. EOAD and LOAD are associated with different patterns of genetic epidemiology.

## 3. Early-Onset Alzheimer's Disease

EOAD is most often caused by rare, highly penetrant mutations in three genes encoding proteins involved in amyloid precursor protein (APP) breakdown and A*β* generation, namely, the* APP *[7],* presenilin 1* (*PSEN1*) [8], and* presenilin 2* (*PSEN2*) [9] genes.

### 3.1. APP Gene

The* APP* gene has 18 exons and encodes a ubiquitously expressed single-pass transmembrane polypeptide of 770 amino acids. A*β* is generated from APP by two endoproteolytic cleavage events catalyzed by *β*-secretase and *γ*-secretase, generating a peptide of 39–43 amino acids in length; the A*β* protein is the major component of the amyloid plaques found in AD brains. The most common form of A*β* is 40 amino acids in length and is called A*β*40; the longer form, A*β*42, is less abundant than A*β*40 but is deposited first and is more amyloidogenic.

Since Goate et al. found the first mutation (V717I) in the* APP* gene in one out of five EOAD families in 1991 [7], 33 different* APP* mutations have been identified in AD patients to date, including 23 missense mutations, nine duplications, and one deletion [10]. Most of the mutations are dominantly inherited. Most of these mutations are positioned in the vicinity of the *β*- and *γ*-secretase cleavage sites (exons 16 and 17 of* APP*); therefore, they influence APP proteolytic processing and/or aggregation. A double point mutation, APP KM670/671NL, identified in two Swedish families, lies at the N-terminus of the A*β* domain adjacent to the *β*-secretase site [11]. Some mutations, such as the Iranian mutation (T714A) [12], Australian mutation (T714I) [13], French mutation (V715M) [14], German mutation (V715I) [15], Florida mutation (I716V) [16], and London mutation (V717I) [7], frame the other end of the A*β* domain, located just distal to the C-terminus of the A*β* domain adjacent to the *γ*-secretase site. Mutations such as the Flemish mutation (A692G) [17], Italian mutation (E693K) [18], Dutch mutation (E693Q) [19], Arctic mutation (E693G) [20], and Iowa mutation (D694N) [21] are located within the A*β* coding sequence. The London mutation (V717I) [7, 22–25], the V717G [26, 27], V717F [25, 28], and V717L [25, 29–31] mutations, the Italian mutation (E693K) [18], Dutch mutation (E693Q) [19], Arctic mutation (E693G) [20], and E693del mutation [32] have been identified in APP residues V717 and E693, making both residues mutation hotspots in the APP gene.

### 3.2. PSEN1 and PSEN2 Gene

In 1995, mutations in the* PSEN1* and* PSEN2* genes, which encode the presenilin 1 and presenilin 2 proteins, respectively, required for *γ*-secretase to produce A*β* from APP, were identified in EOAD families [8, 9, 33]. To date, approximately 200 different AD-related* PSEN1* mutations and 22 AD-related* PSEN2* mutations have been detected (http://www.molgen.vib-ua.be/ADMutations/) [10, 34–38]. The majority of mutations are missense mutations, but small deletions and insertions have also been reported. Mutations in* PSEN1* and* PSEN2* cause amino acid sequence changes throughout the protein with some clustering within the transmembrane domains and the hydrophilic loops. Mutations in exons 5, 6, 7, and 8 account for >70% of all identified mutations in* PSEN1* (http://www.molgen.vib-ua.be/ADMutations/) [10]. Five different mutations of* PSEN1* residue 143 (I143V/F/N/T/M, encoded by exon 5) [39–43] have been identified, making I143 residue a mutation hotspot. The presenilins act as aspartyl proteases that carry out *γ*-secretase cleavage of APP to produce A*β*. Therefore, mutations in the* PSEN1* and* PSEN2* genes increase the ratio of A*β*42 to A*β*40.

*PSEN1* mutations account for the majority (78%) of the EOFAD mutations identified, followed by* APP* (18%), with* PSEN2* mutations found in only a few families (4%). Together, the mutations in these three genes are thought to be responsible for 30–50% of autosomal dominant AD cases and about 0.5% of AD cases in general (http://www.molgen.ua.ac.be/ADMutations) [10]. Although mutations in these genes are a rare cause of AD, the identification of these genes and mutations has been extremely important to the recent progress in the understanding of the biology of AD.

## 4. Pathophysiology

In 1984, Glenner first proposed that cerebral A*β* drives all subsequent pathology [44]. This central thesis was later reinterpreted and reported as the amyloid cascade hypothesis of AD, which maintains that the accumulation of A*β* is the primary driver of AD-related pathogenesis, including neurofibrillary tangle formation, synapse loss, and neuronal cell death [45–47]. Although the amyloid cascade is only one possible mechanism proposed for AD and amyloid alone is probably not sufficient to cause AD, the association of* APP*,* PSEN1*, and* PSEN2 *with amyloid synthesis and its processing indicates that the amyloid cascade theory plays an important role in EOAD. According to the amyloid cascade hypothesis, AD is a consequence of an imbalance between production and deposition of A*β*.

The AD-linked APP mutations located near the *β*- and *γ*-secretase cleavage sites serve to increase A*β*42 production. The Swedish mutation, lying immediately before the beginning of the A*β* peptide sequence, affects the efficiency of *β*-secretase cleavage and increases the amount of A*β* peptide produced by 2-3 fold [11]. APP mutations at the C-terminal end of A*β* alter the activity of *γ*-secretase cleavage and shift proteolysis to produce more A*β*42, resulting in an increased A*β*42/A*β*40 ratio but not an increase in the total amount of A*β* peptides formed [48]. The Arctic mutation (E693G) is located within A*β* dominant; therefore, rather than altering the amount of A*β* formed or the A*β*40/A*β*42 ratio, it increases the rate of aggregation of mutant peptide, suggesting that* APP* mutations can result in A*β* peptides with altered aggregation properties [20]. The amyloid hypothesis is further supported by the opposite situation, in which an APP mutation reducing the amount of amyloid formation in fact protects against AD [49].

Both presenilins 1 and 2 are part of the *γ*-secretase complex; therefore, they are functionally involved in the *γ*-secretase-mediated proteolytic cleavage of APP. *γ*-Secretase containing mutation-altered presenilin still catalyzes cleavage of APP, but the proteolytic site is altered; therefore, it causes either an increase in A*β*42 levels or a decrease in A*β*40 levels, leading to an increase in the A*β*42/40 ratio. The relative increase in A*β* promotes the aggregation of the peptide into oligomers and ultimately amyloid fibrils [46, 50–52].

## 5. Genotype-Phenotype Correlations

The clinical features of EOAD are heterogeneous, most probably due to different genetic mutations and epigenetic factors. Although the phenotypes associated with some mutations have been well characterized, significant phenotypic heterogeneity also exists both between families sharing a common mutation and within the affected individuals of a single family.

### 5.1. Clinical Features of EOAD with APP Mutations

Patients with APP mutations tend to present with symptoms between the ages of 40 and 65 years and have disease duration of 9–16 years. Among the APP missense mutation families reported, the phenotypes of the Flemish mutation (A692G), Dutch mutation (E693Q), Arctic mutation (E693G), Iowa mutation (D694N), and London mutation (V717I) are the most well studied and described. The phenotype is heterogeneous among these mutations. Mutations at APP residue V717, including the London mutation (APP V717I) [7, 22–25], V717G [26, 27], V717F [25, 28], and V717L [25, 29–31], demonstrate a similar phenotype with early impairment of episodic memory, dyscalculia, lack of insight, and prominent myoclonus and seizures [53]. Clinically, patients with the Flemish mutation (APP A692G) present, in their 40s, either with symptoms related to cerebrovascular events or with cognitive dysfunction; neuropathologically, massive amyloid accumulation in brain vessel walls but no neurofibrillary tangles is seen in these patients [17, 19, 54, 55]. Carriers of the Dutch mutation (APP E693Q) are characterized clinically by focal symptoms related to recurrent cerebrovascular events, usually followed by dementia, in their 40s and 50s, and pathologically by cerebral amyloid angiopathy with rare amyloid plaque pathology [19, 56, 57]. Despite the presence of marked amyloid angiopathy with abundant amyloid plaques and neurofibrillary tangles on pathological investigation, patients with the Arctic (APP E693G) mutation demonstrate a purely cognitive phenotype in their 50s [20, 58, 59]. A similar pathological feature was found in individuals with the Iowa (APP D694N) mutation: the clinical feature of the mutation carriers is progressive dementia with speech impairment without any apparent focal symptoms of cerebrovascular events [21, 60].

It is now known that duplications of the* APP* gene can give rise to familial AD. The characteristic features associated with APP duplication include a high frequency of seizures with early progressive impairment of episodic memory and prominent amyloid angiopathy leading to frequent intracerebral hemorrhage [61–65].

### 5.2. Clinical Features of EOAD with PSEN1 Mutations

EOAD patients with* PSEN1* mutation typically have an early age of onset, being an average of 8.4 years earlier than the age of onset in* APP* mutation carriers (average 42.9 versus 51.3 years) and 14.2 years earlier than the age of onset in* PSEN2* mutation carriers (average 57.1 years) [10]. Patients with very early-onset Alzheimer's disease (VEOAD) usually have an age of onset before the age of 35 years. A study of 101 VEOAD cases from 1934 to 2007 found a* PSEN1* mutation or linkage to chromosome 14 in all cases for which genetic analysis was performed, suggesting that* PSEN1* mutations may be responsible for all reported cases of VEOAD [66].

*PSEN1* mutation carriers predominantly showed impairment of memory and multiple cognitive domains [67, 68]. In addition to cognitive symptoms, some unusual clinical features may be present early in the course of the disease, in particular myoclonus and seizures [69]. Despite almost all of these symptoms having been reported in sporadic AD patients, extrapyramidal signs, behavioral and psychiatric symptoms (agitation, depression, delusion, and hallucinations), aphasia, and cerebellar signs are significantly more frequent in* PSEN1* mutation carriers [70, 71].

The characteristic phenotype associated with a* PSEN1* mutation probably represents the syndrome of “variant AD,” which is most often associated with mutations around exon 8 and characterized by early-onset familial dementia and spastic paraparesis [72, 73]. The spastic paraparesis may occur simultaneously with cognitive deficits or may predate them by several years. The patients typically present with insidiously progressive impaired gait and mild weakness in the lower limbs, with signs of hyperreflexia [73, 74]. The mean age of onset of “variant AD” ranges from 27 to the mid-50s, with the most typical age of onset being at the earlier ages in the spectrum [71, 73]. Therefore, it is not surprising that many VEOAD cases have been reported to demonstrate this syndrome [75, 76].

### 5.3. Clinical Features of EOAD with PSEN2 Mutations

Of the families identified with* PSEN2* mutations, Volga German pedigrees with the N141I mutation are the largest and most well-studied group. Compared with* PSEN1* mutation carriers, carriers of* PSEN2* mutations have a later age of onset. The age of onset associated with mutations in* PSEN2* is typically in the 50s, but there is a wide range from 39 to 75 years. The average disease duration is 10.6 years, longer than that in patients with* PSEN1* mutations (8.4 years) but similar to that in sporadic late onset AD patients (10.6 years). Early and progressive defects in memory and executive functions are common with relative sparing of naming. Seizures (30%) are common in individuals with* PSEN2* mutations. Neuropathological changes are those of typical AD with extensive neuritic plaque formation and neurofibrillary tangle accumulation. Amyloid angiopathy may be prominent and even lead to hemorrhagic stroke [77].

## 6. Late-Onset Alzheimer's Disease

For most cases of LOAD, the risk of developing AD is assumed to be determined by genetic variants combined with lifestyle and environmental exposure factors. The *ε*4-allele of the apolipoprotein E gene (*APOE*) on chromosome 19q13.2 is the only well-established genetic risk factor for LOAD [78]. There are three common alleles of ApoE in humans (*ε*2, *ε*3, and *ε*4) that differ in sequence by only one amino acid at either position 112 or 158 of the protein. One copy of the *ε*4-allele of* APOE* increases AD risk by 4-fold and two copies increase the risk by 12-fold; by contrast, the *ε*2-allele of* APOE* is protective against AD [79]. APOE *ε*4-alleles are also associated with a dose-dependent decrease in age at onset (∼5 years/*ε*4-allele) in both sporadic and familial AD cases [80, 81]. Functionally, ApoE maintains lipoprotein metabolism and transport, but it is believed to play a role in the clearance of cerebral A*β* in AD pathogenesis. A*β* deposits are more abundant in *ε*4-positive than in *ε*4-negative cases [82]. In addition, ApoE4 is associated with other factors that may contribute to AD pathology, including low glucose usage, mitochondrial abnormalities, and cytoskeletal dysfunction [83]. Furthermore, the presence of this allele is associated with memory impairment, MCI, and progression from MCI to dementia [84].* APOEε*4 has been suggested to account for as much as 20–30% of AD risk.

Since the report of an association of LOAD with* APOE*, many hundreds of studies have been performed to look for AD susceptibility genes, but few reported genetic associations have been replicated across studies. The main reason for this is that other genetic risk factors have much smaller effect sizes than does* APOE*. As a result, the sample sizes used in the earlier studies were too small to have the power to detect these genes. During the last few years several technological advances have transformed the landscape regarding the genetics of common complex traits such as AD. The first of these was the development of genome-wide arrays that allowed simultaneous screening of the entire human genome in thousands of samples for novel AD loci. Since 2007, dozens of GWAS of thousands of samples from AD patients and nondemented elderly controls have been performed, resulting in the identification of a number of new genetic loci with genome-wide significance (<5 × 10^−8^) [85–109]. Some of these foci have been identified in more than two GWAS and have compelling evidence supporting an association with LOAD, including* clusterin *(*CLU*) [95, 96, 99, 107],* complement component receptor 1* (*CR1*) [95, 104–106], and* phosphatidylinositol binding clathrin assembly protein* (*PICALM*) [96, 99, 107, 108],* bridging integrator 1* (*BIN1*) [99, 101, 104–107],* sialic acid binding Ig-like lectin* (*CD33*) [90, 105–107],* CD2-associated protein* (*CD2AP*) [105, 106],* membrane spanning 4A gene cluster* (*MS4A6A/MS4A4E*) [105–108],* ephrin receptor A1* (*EPHA1*) [105–107], and* ATP-binding cassette transporter* (*ABCA7*) [105–107, 109]. Recently, a rare susceptibility variant (rs75932628) in the* triggering receptor expressed on myeloid cells 2* (*TREM2*) gene was identified to be associated with LOAD [110, 111].

The search for additional genetic risk factors requires large-scale meta-analysis of GWAS data to increase statistical power. Very recently, a large, two-stage meta-analysis of AD GWAS in individuals associated with European ancestry identified 11 novel genetic loci for LOAD in addition to the already known genes:* ABCA7*,* APOE*,* BIN1*,* CLU*,* CR1*,* CD2AP*,* EPHA1*,* MS4A6A-MS4A4E,* and* PICALM* genes [112]:* HLA-DRB5–HLA-DRB1* (encoding major histocompatibility complex, class II, DR*β*5 and DR*β*1),* SORL1 *(encoding sortilin-related receptor, L(DLR class) 1),* PTK2B* (encoding protein tyrosine kinase 2*β*),* SLC24A4-RIN3 *(encoding solute carrier family 24 (sodium/potassium/calcium exchanger), member 4),* ZCWPW1* (encoding zinc finger, CW type with PWWP domain 1),* CELF1 *(encoding CUGBP, Elavlike family member 1),* NME8*,* FERMT2 *(encoding fermitin family member 2),* CASS4* (encoding Cas scaffolding protein family member 4),* INPP5D *(encoding inositol polyphosphate-5-phosphatase, 145 kDa), and* MEF2C* (encoding myocyte enhancer factor 2). This brought the number of genetic loci associated with LOAD up to 22 [112].

Rare highly penetrant mutations were also reported in LOAD. Two rare mutations in the* ADAM10* gene, Q170H and R181G, are identified in 7 out of 1000 LOAD families. Both mutations are located in the prodomain region and dramatically impair the ability of ADAM10 to cleave APP at the *β*-secretase site of APP in vitro and in vivo [113]. These findings highlight the importance of the employment of whole genome or whole exome sequencing to identify rare variants causing LOAD, in addition to common variants, in future genetic studies [113].

Although the total number of AD risk genes remains elusive, there is good evidence suggesting that, in combination, they have a substantial impact on AD predisposition. The new risk alleles have a much smaller effect on AD susceptibility than does* APOE*ε*4*. Estimates of the population-attributable fractions or preventive fractions for these new loci are in the range 1–8.1%. The cumulative population-attributable fraction effect and population preventive fraction effect for non-*APOE* candidate genes are estimated to be 31.7% and 30.4%, respectively, while the population-attributable fraction for* APOE*ε*4* is about 27.3% [112]. However, the actual effect sizes of the new loci may be much smaller because of the “winner's curse,” a bias that arises in GWAS studies because of sampling variation and depends strongly on the power of the initial test for an association (since the new loci are only weakly associated with AD, the power to detect association is low and the odds ratios for the new loci are usually overestimated) [114].

**Figure 1:**
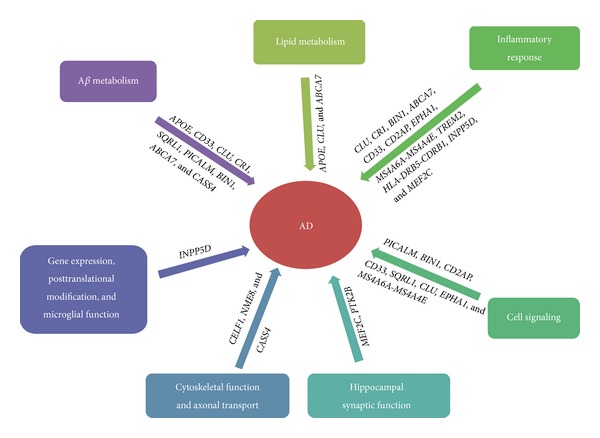
Potential pathways of susceptibility genes involved in the pathogenesis of AD.

These AD risk genes affect various pathways, roughly falling into four pathways: A*β* metabolism, lipid metabolism, immune and complement system/inflammatory response, and cell signaling (Figure 1). Eleven loci (*CLU*,* CR1*,* BIN1*,* ABCA7*,* CD33*,* CD2AP*,* EPHA1*,* MS4A6A-MS4A4E, TREM2*,* HLA-DRB5-CDRB1*,* INPP5D*, and* MEF2C*) probably affect inflammatory processes; nine loci (*APOE*,* CD33*,* CLU*,* SQRL1*,* CR1*,* PICALM*,* BIN1*,* ABCA7*, and* CASS4*) are involved in A*β* metabolism; eight loci (*PICALM*,* BIN1*,* CD33*,* CD2AP*,* SQRL1, CLU, EPHA1, *and* MS4A6A-MS4A4E*) are involved in processes at the cell membrane, including endocytosis; and three loci (*APOE*,* CLU,* and* ABCA7*) are involved in lipid biology [112, 115]. Some of these newly identified loci also suggest the existence of new pathways underlying AD, probably including hippocampal synaptic function (*MEF2C* and* PTK2B*), cytoskeletal function and axonal transport (*CELF1*,* NME8,* and* CASS4*), regulation of gene expression and posttranslational modification of proteins, and microglial and myeloid cell function (*INPP5D*) [112]. Therefore, the discoveries of these new genes open new avenues to study the pathogenesis of AD. By targeting the specific components of the pathways these gene products are involved in, novel directions for drug discovery and effective treatment may be available [114].

Although up to 80% of the AD risk is supposed to be attributable to genetic factors, all of the known LOAD genes (including* APOE* and new ones) account for about 60% of the total genetic variance, indicating that additional risk genes for LOAD remain to be identified.

## 7. Clinical Implications

### 7.1. Clinical Genetic Testing

Genetic testing of patients with early-onset dementia and a positive family history can be a valuable tool for identifying causative mutations, establishing the correct diagnosis, excluding some differential diagnoses, and offering at-risk relatives the option of predictive testing [76, 116]. In addition, by performing genetic testing, the physician will be able to select patients with the same underlying pathogenesis for therapeutic trials [117]. Because mutations in* PSEN1* are the most frequent cause of EOFAD, it is rational to screen for* PSEN1* mutations first, particularly if the patients have early age of onset, followed by* APP *and* PSEN2* mutations [118]. If sequencing of all three genes is normal for a Mendelian family, then screening for duplication of the* APP* gene should also be considered [65, 117]. As discussed above, a few phenotypic clues can help prioritize mutation screening. Familial AD patients with spastic paraparesis are likely to have a* PSEN1* mutation while familial AD patients with cerebral hemorrhage caused by cerebral amyloid angiopathy may have* APP* mutations [118]. Mutations in these genes may occasionally be identified in AD patients with no family history, especially those with an age of onset of 40 years or younger. These cases may be “true” sporadic patients withde novo mutations or “apparent” sporadic patients with no obvious family history because of a small family size, early deceased parents, incomplete penetrance, or paternity issues. Therefore, genetic testing in EOAD patients with no family history can be useful in establishing the diagnosis [117]. An algorithm for genetic testing in patients with EOAD has been proposed elsewhere [117]. It should be kept in mind that some variants might only be polymorphisms with no clinical significance rather than pathogenic mutations, especially for genetic changes that have not been reported in the past. Guerreiro et al. proposed a scale for grading mutations as not pathogenic, possibly pathogenic, probably pathogenic, and definitely pathogenic, on the basis of segregation within the family, its frequency in clinically normal individuals, and functional studies in model systems [119].

### 7.2. Presymptomatic Genetic Counselling

With respect to asymptomatic relatives of an EOFAD case with a pathogenic mutation, the availability of genetic testing and counselling creates both advances and dilemmas. Common reasons for testing include concerns about the early symptoms of dementia, reproductive planning, and the psychological burden of the uncertainty about their future [120, 121]. Furthermore, identified asymptomatic mutation carriers might also have the opportunity to join treatment trials for genetic at-risk groups, such as the Alzheimer's Prevention Initiative and Dominantly Inherited Alzheimer Network initiatives [118]. On the other hand, genetic testing may trigger potential risk of emotional stress, such as depression, anxiety, or even suicide [76]. However, two independent studies have shown that, in the majority of asymptomatic individuals tested using a standardized counseling protocol, the testing was beneficial and the patients demonstrated effective coping skills without negative psychological reactions during several months of follow-up [120, 121]. Although genetic variations other than these known genes also contribute to AD risk, among which variants in* ApoE* and* TREM2* gene have the greatest effect, genetic testing for* ApoE* or* TREM2* genotypes is not recommended for asymptomatic individuals [118]. Guidelines and recommendations on genetic counseling procedures in asymptomatic AD have been published previously [116].

### 7.3. Gene Therapy

To date, no disease-modifying treatment for AD is yet available, although several disease-modifying clinical trials are ongoing. Based on the amyloid cascade hypothesis, in the majority of AD cases, the accumulation of A*β* is a result of an imbalance between A*β* generation and clearance. Thus, a gene therapy approach involving A*β*-degrading enzymes would be a possible alternative strategy [122]. Gene delivery of nerve growth factor (NGF) [123], brain-derived neurotrophic factor (BDNF) [124], neprilysin [125], APOE [126], ECE [127], and cathepsin B [128] has been studied extensively in several AD animal models with promising results, and the first clinical trial using ex vivo gene delivery has been completed, with result indicating amelioration of AD pathogenesis [129]. The first human clinical trial based on rAAV-mediated NGF gene therapy has been initiated [130]. Downregulating APP [131] and *β*-site APP cleaving enzyme 1 (BACE1) [132] levels in vivo by means of siRNA-mediated knockdown has also produced some very promising results. Therefore, gene therapy appears to have the potential to become a disease-modifying treatment for AD [122].

## 8. Perspectives

There have been huge advances in our understanding of the genetics of AD over the last few years. The discovery of the three EOAD-related genes,* APP*,* PSEN1,* and* PSEN2*, has improved our knowledge of the physiopathology of AD. Ongoing and future large-scale genome-wide association studies and next-generation whole genome or whole exome sequencing hold the promise of unraveling the complexities of the genetic architecture of this disease. This should lead to identification of novel targets for genetic testing, as well as developing preventative and curative therapies for AD.

## Supplementary Material

Overview of GWAS in AD.
