# Supplementary material for: Clinical Genetics of Alzheimer's Disease
Source: Biomed Res Int. 2014 May 13;2014:291862. doi: 10.1155/2014/291862 (PMC4052685; doi:10.1155/2014/291862)
Supplement: Supplementary file 1 — Overview of GWAS in AD. [file 291862.f1.pdf]

Table 1: Overview of all published Genome-wide Association Studies in Alzheimer's disease

Study	Ethnic	SNPS	Sample size	Genes identified
Grupe, 2007[85]	USA, UK	17,343	Stage 1: 380 AD cases; 396 controls<br><br>Stage 2: 1,428 AD cases; 1,666 controls	TOMM40, APOE, GALP, TNK1, APOC2, PCK1, LMNA, PGBD1, LOC651924, THEM5, MYH13, BCR, CTSS, UBD
Coon, 2007[86]	USA, Netherlands	502,627	664 AD cases; 422 controls	APOE
Reiman, 2007[87]	USA, Netherlands	502,627	Stage 1: 446 AD cases; 290 controls<br><br>Stage 2: 415 AD cases; 260 controls	GAB2
Li, 2008[88]	Canada	469,438	Stage 1: 753 AD cases; 736 controls<br><br>Stage 2: 418 AD cases; 249 controls	APOE, GOLM1, GWA_15q21.2,<br><br>GWA_9p24.3
Abraham, 2008[89]	USA, Europe	561,494	Stage 1: 1,082 AD cases; 1,239 controls<br><br>Stage 2: 1,400 controls	APOE, LRAT
Bertram, 2008[90]	USA	484,522	Stage 1: 941 AD cases; 404 controls<br><br>Stage 2: 1,767 AD cases; 838 controls	APOE, ATXN1, CD33, GWA_14q31.2
Poduslo, 2009[91]	USA	489,218	Stage 1: 9 AD cases; 10 controls<br><br>Stage 2: 199 AD cases; 225 controls	TRPC4AP
Beecham, 2009[92]	USA	555,000	Stage 1: 492 AD cases; 496 controls<br><br>Stage 2: 238 AD cases; 220 controls	APOE, FAM113B
Carrasquillo, 2009[93]	USA	313,504	Stage 1: 844 AD cases; 1,255 controls<br><br>Stage 2: 1,547 AD cases; 1,209 controls	APOE, PCDH11X
Potkin, 2009[94]	USA	516,645	172 AD cases; 209 controls	APOE, ARSB, CAND1, EFNA5, MAGI2, PRUNE2, TOMM40
Harold, 2009[96]	USA, Europe	529,205	Stage 1: 3,941 AD cases; 7,848 controls<br><br>Stage 2: 2,023 AD cases; 2,340 controls	APOE, CLU, PICALM
Lambert, 2009[95]	Europe	537,029	Stage 1: 2,032 AD cases; 5,328 controls<br><br>Stage 2: 3,978 AD cases; 3,297 controls	APOE, CLU, CR1
Seshadri, 2010[99]	USA, Europe	2,540,000	Stage 1: 3,006 AD cases; 14,642 controls<br><br>Stage 2: 2,032 AD cases; 5,328 controls	APOE, BIN1, CLU, EXOC3L2, PICALM
Bettens, 2010[98]	Belgium	4	1,078 AD cases and 652 controls	ATXN1
Laumet, 2010[97]	French	526	2,032 AD cases, 5,328 controls	TFAM, SORL1, CHRNB2, SORCS1, DAPK1, MTHFR, GWA14q32.13, BDNF, NEDD9, and CH25H

Naj, 2010[100]	USA	483,399	Stage 1: 931 AD cases; 1,104 controls<br><br>Stage 2: 1,338 AD cases; 2,003 controls	APOE, MTHFD1L
Lee, 2011[101]	Caribbean Hispanic	627,380	549 AD cases; 544 controls	CLU, PICALM, BIN1, CUGBP2, loci on 2p25.1; 3q25.2; 7p21.1; 10q23.1
Sherva, 2011[102]	Israel	2,540,000	124 AD cases; 142 controls	AGPAT1, ATP6V0A4, GLOD4, RGS6, TMEM132C
Wijsman, 2011[103]		565,336	Stage 1: 1,848 AD cases; 1,991 controls<br><br>Stage 2: 617 AD cases; 573 controls	APOE, BIN1, CLU, CR1
Hollingworth, 2011[106]	USA, Europe	496,763	Stage 1: 6,688 AD cases; 13,685 controls<br><br>Stage 2: 4,896 AD cases; 4,903 controls<br><br>Stage 3: 8,286 AD cases; 21,258 controls	ABCA7, BIN1, CD2AP, CD33, CR1, EPHA1, MS4A4E, MS4A6A
Naj, 2011[105]	USA	2,324,889	Stage 1: 8,309 AD cases; 7,366 controls<br><br>Stage 2: 3,531 AD cases; 3,565 controls	APOE, BIN1, CD2AP, CD33, CLU, CR1, EPHA1, MS4A4A
Hu, 2011[104]	USA	509,376	step 1: 1831 AD cases and 1764 controls<br><br>step 2: 751 AD cases and 751 controls	CR1, CLU
Logue, 2011[107]	African Americans	>600,000	513 AD cases and 496 controls	PVRL2, CLU, PICALM, BIN1, EPHA1, MS4A, ABCA7, and CD33
Kamboh, 2012[108]	USA	1,016,423	Stage 1: 1,440 AD cases and 1,000 controls<br><br>Stage 2: 2,727 cases and 3,336 controls	APOE, PICALM, BIN1, ABCA7, EPHA1, MS4A/MS4A6E, PPP1R3B
Shi, 2012[109]	USA, UK	600,489	1047 AD cases; 338 controls	APOE
Reitz, 2013[110]	African American		1,968 AD cases; 3,928 controls	ABCA7, intergenic locus on 5q35.2
Jonsson, 2013[112]	Icelander,<br><br>Germany, Netherlands, Norway	191,777	Stage 1: 1,092 AD cases; 110,050 controls<br><br>Stage 2: 2,037 AD cases; 9,727 controls	TREM2
Guerreiro, 2013[111]	European North American		Stage 1: 3,550 AD cases; 1,107 controls<br><br>Stage 2: 1,887 AD cases;4,061 controls	TREM2
Lambert JC, 2013[113]	Europe	7,055,881<br><br>11,632	Stage 1: 17,008 AD cases; 37,154 controls<br><br>Stage 2: 8,572 AD cases; 11,312 controls	APOE, ABCA7, BIN1, CD33, CLU, CR1,<br><br>CD2AP, EPHA1, MS4A6A-MS4A4E, PICALM, HLA-DRB5–HLA-DRB1, PTK2B, SORL1, SLC24A4-RIN3, ZCWPW1, CELF1, FERMT2, CASS4, INPP5D, MEF2C
